# Contrast vector imaging for differential diagnosis of focal liver lesions: Analysis of tumoral vascular structures and flow characteristics

**DOI:** 10.1371/journal.pone.0314263

**Published:** 2024-12-03

**Authors:** Jeongin Yoo, Jeong Min Lee, Ijin Joo, Jeong Hee Yoon

**Affiliations:** 1 Department of Radiology, Seoul National University Hospital, Seoul, South Korea; 2 Department of Radiology, Seoul National University Hospital, Seoul National University College of Medicine, Institute of Radiation Medicine, Seoul National University Medical Research Center, Seoul, South Korea; 3 Department of Radiology, Seoul National University Hospital, Seoul National University College of Medicine, Seoul, South Korea; Universitatsklinikum Leipzig, GERMANY

## Abstract

**Purpose:**

To assess the efficacy of contrast vector imaging (CVI) in detecting tumoral vascular structures and flow characteristics of focal liver lesions (FLLs) for differential diagnosis.

**Materials and methods:**

In this prospective study, 65 participants with FLLs underwent CEUS using SonoVue with high-frame-rate imaging technique between July 2019 and October 2020. CVI was obtained by post-processing arterial cine imaging of CEUS. Tumoral vascular structures, velocity histogram, and mean velocities were compared among hepatocellular carcinoma (HCC), non-HCC malignancies, and benign tumors using the Chi-square and Kruskal-Wallis tests, respectively. The areas under the receiver operating characteristic curve (AUC) of CEUS in determining HCC probability was compared to that of CEUS with CVI using a z-test.

**Results:**

CVI was technically successful in 52 of 65 (80%) participants (19 HCCs, 13 non-HCC malignancies, and 20 benign tumors). The detectability of tumoral vascular structures was significantly higher in CEUS with CVI, compared to CEUS alone (46.2% [24/52] vs. 100.0% [52/52], p<0.001). On CEUS with CVI, complex intratumoral and peripheral vessels were frequent in HCCs (100% of HCCs, 46.2% of non-HCC malignancies, and 70.0% of benign tumors), while detour vessels were frequent in non-HCC malignancies (none of HCCs, 53.8% of non-HCC malignancies, and 10.0% of benign tumors) (p<0.001). The mean velocity of HCC (26.3 mm/s) was the highest, while that of non-HCC malignancy (20.6 mm/s) was the lowest (p<0.001). CEUS with CVI showed higher AUC, compared to CEUS in both reviewers (0.851 vs. 0.963, p = 0.005 for reviewer 1; 0.853 vs. 0.982, p = 0.023 for reviewer 2).

**Conclusion:**

CEUS with CVI better visualized vascular structures and flow characteristics of FLLs, and showed better diagnostic performance in determining HCC probability than CEUS.

## Introduction

Contrast-enhanced ultrasound (CEUS) is currently recommended as the first-line imaging technique for the characterization of incidentally detected, indeterminate focal liver lesions (FLLs) in patients with a non-cirrhotic liver and without a history or clinical suspicion of malignancy [1]. The real-time functionality of CEUS offers enhanced sensitivity in uncovering the dynamic enhancement patterns of FLLs compared to contrast-enhanced computed tomography (CT) or magnetic resonance imaging (MRI) [1]. Moreover, CEUS has a high diagnostic accuracy in distinguishing hepatocellular carcinoma (HCC) from other malignancies in the cirrhotic liver [2]. Recently, CEUS is recommended as a primary [3] or secondary [4–6] imaging modality for diagnosing HCC in at-risk patients according to major guidelines. Although the typical enhancement features of HCC are “arterial phase hyperenhancement” and “mild and late washout” [2], 5%–41% of HCCs, may show sustained enhancement during the portal venous and late phases, and therefore, sensitivity for HCC diagnosis is around 70% while specificity is higher than 95% [2, 7]. In addition, the diagnosis of most benign focal liver lesions presents a challenge owing to complete hyper- or isoenhancement during the portal venous and late phases [8].

Although CEUS may show peculiar patterns of the vascular structures of some FLLs, such as spoked wheel arteries of focal nodular hyperplasia (FNH) or basket pattern or chaotic vessels of HCC, observation of these vascular structures is limited to a few seconds after the arrival of contrast bubbles [1, 9, 10]. Furthermore, microflow imaging technique based on the maximum intensity capture of microbubbles from consecutive low-power images, has been shown to better depict the vascular architecture of lesions than conventional CEUS, but it is limited by the challenge of image registration over multiple images, or loss of spatial resolution related to maximum intensity capture [8, 11].

Post-processing contrast vector imaging (CVI) (Canon Medical Systems; Tochigi, Japan) is a technique for quantitative assessment of blood flow in tissues or tumors [12]. This entails implementing a tracking method on unprocessed CEUS data at a frame rate of 32–64 frames/sec, which is approximately 2–4 times faster than conventional contrast-specific harmonic imaging techniques [12]. Consequently, it holds the potential to enhance the visualization of peripheral and internal tumor vessels, offering superior spatial and temporal resolution. Our hypothesis is that CVI’s bubble tracking ability may reveal unique vascularization patterns in FLLs, leading to improved characterization of FLLs.

Therefore, the purpose of this study was to assess the efficacy of post-processing CVI in detecting tumoral vascular structures and flow characteristics of focal liver lesions (FLLs) for differential diagnosis.

## Materials and methods

This prospective study was approved by our Institutional Review Board, and written informed consent was obtained from all patients. A part of the patients (30 patients) was reported in a previous study [12] which focused on the technical feasibility of CVI in FLLs.

### Patients

**Fig 1:**
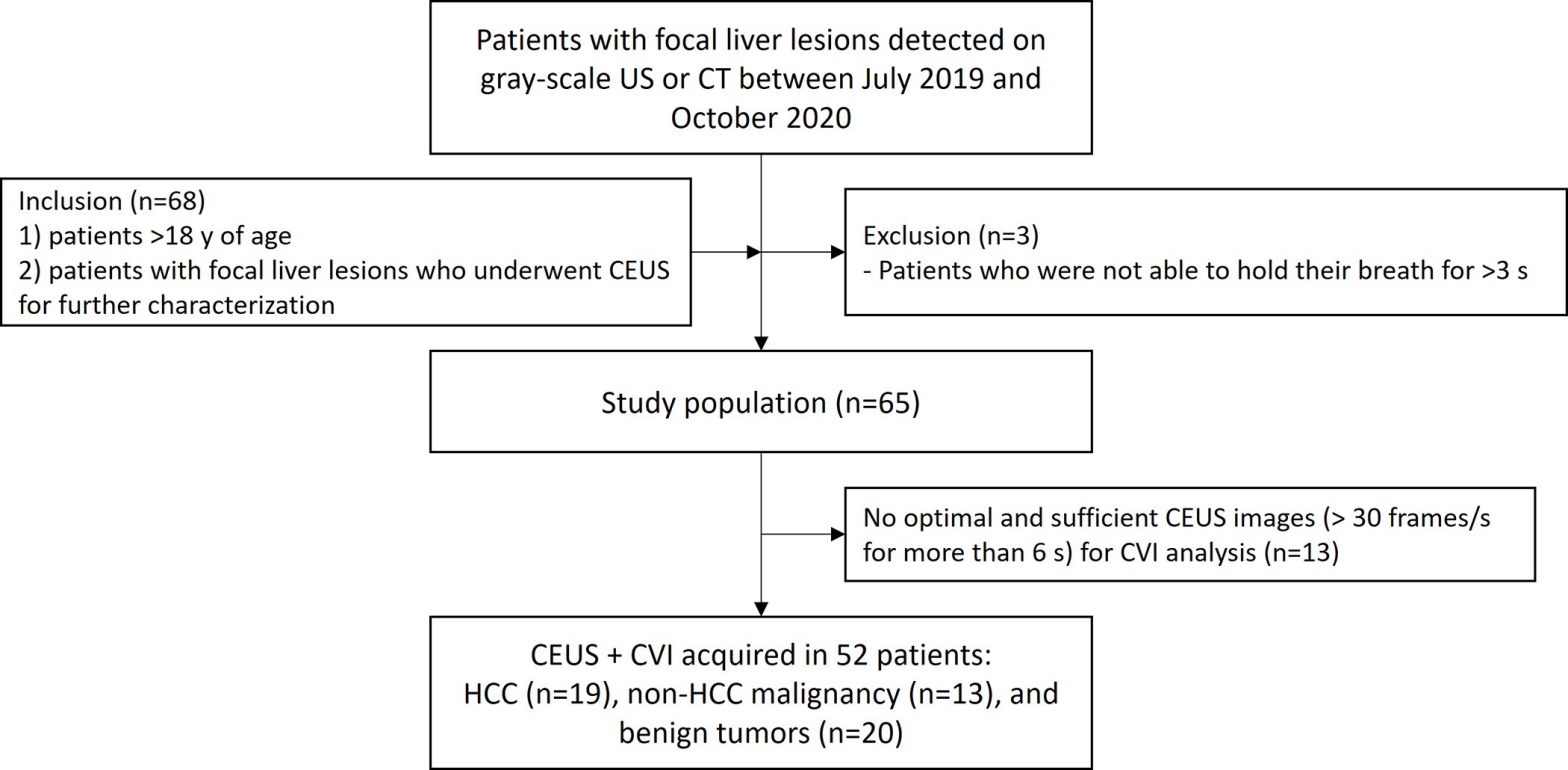
Patient enrollment process.

Between July 18^th^ 2019 and October 31^st^ 2020, we screened study candidates who were referred to department of radiology for CEUS examination for evaluation of FLL or for percutaneous biopsy. The inclusion criteria were: 1) >18 years of age; 2) patient who have FLLs detected on gray-scale US or indeterminate lesions on contrast-enhanced CT; or 3) patients who have inconspicuous tumor margin on B mode US before percutaneous biopsy Fig 1). Exclusion criteria were 1) poor sonic window on B-mode US; and 2) patients who were not able to hold their breath for >3 seconds.

### CEUS protocol

Patients fasted for at least 6 hours before the examination. CEUS was performed by one of two radiologists (either J.M.L. or J.Y. with 16 and 2 years of experience, respectively) using a US system (Aplio i800; Canon Medical Systems, Tochigi, Japan) and a wideband convex i8CX1 multi-frequency probe. CEUS images were obtained in contrast-specific ultrasound mode with the following parameters: contrast harmonic frequency, 3.0 MHz; mechanical index, 0.09; dynamic range, 60 dB; gain, 76 dB; and frame rate, 32 frames/sec. With a multi-beam receiver and multi-harmonic compounding technique, high-temporal-resolution arterial phase imaging was obtained for detecting and tracing contrast bubbles [12]. Immediately after intravenous bolus administration of 2.4 mL of SonoVue (Bracco Imaging S.p.A., Milan, Italy) via the antecubital vein, followed by 5 mL of normal saline, continuous CEUS images of the target lesion were obtained under breath-hold for 7–10 sec during the arterial phase (approximately 10–20 sec after contrast agent injection when the contrast arrived at the lesion), followed by interval scans of 5 sec duration in every 30 sec for 5 min.

### Post-processing CVI

To post-process CVI, we used online software of the same clinical US system (Aplio i800) used for the CEUS examination. The US system traces and automatically matches the speckle pattern of each template image between frames [13]. There are seven display types: arrival time, direction, direction center, velocity, velocity variance, trace, and arrow [12] (S1 Fig in S1 File). The trace and arrow visualized all the bubbles that were detected, while the other five elucidated the contiguous movement of bubbles. If a contrast bubble was detected on a certain frame and did not move or disappear in the following frame, it could not be visualized via the latter five types. Each display type was color-coded to provide information on the detected bubbles in the form of either velocity or direction. In addition to the velocity map, the mean velocity of the region-of-interest and histogram analysis of velocity were automatically provided. It takes an approximately 5 min to generate post-processing CVI in each case.

### Image analysis

The same radiologist who performed CEUS and another aforementioned radiologist independently reviewed CEUS with CVI. They were blinded to the pathologic diagnosis of FLLs. Technical failure of CVI was defined when CVI maps could not be post-processed due to failure of obtaining sufficient CEUS images (> 30 frames/sec for more than 6 s). Tumoral vascular structures were evaluated on CEUS alone and CEUS with CVI, respectively, with a 1-month interval to reduce recall bias. Arterial enhancement patterns on CEUS with CVI were categorized into diffuse staining, peripheral rim, and peripheral globular [9]. Washout patterns were evaluated on CEUS images, and the washout time and degree were assessed and classified as early, late mild, marked, and absent [3]. Early washout was defined as washout that was unequivocally detectable earlier than 60 seconds after contrast injection, while late washout referred to washout that was unequivocally detectable at 60 seconds or later [14]. Washout was considered mild when a nodule is hypoenhancing relative to the liver, whereas marked washout was defined as a nodule appearing almost black with minimal internal enhancement or is seen as a punched-out lesion within 2 minutes after contrast injection [14].

**Fig 2:**
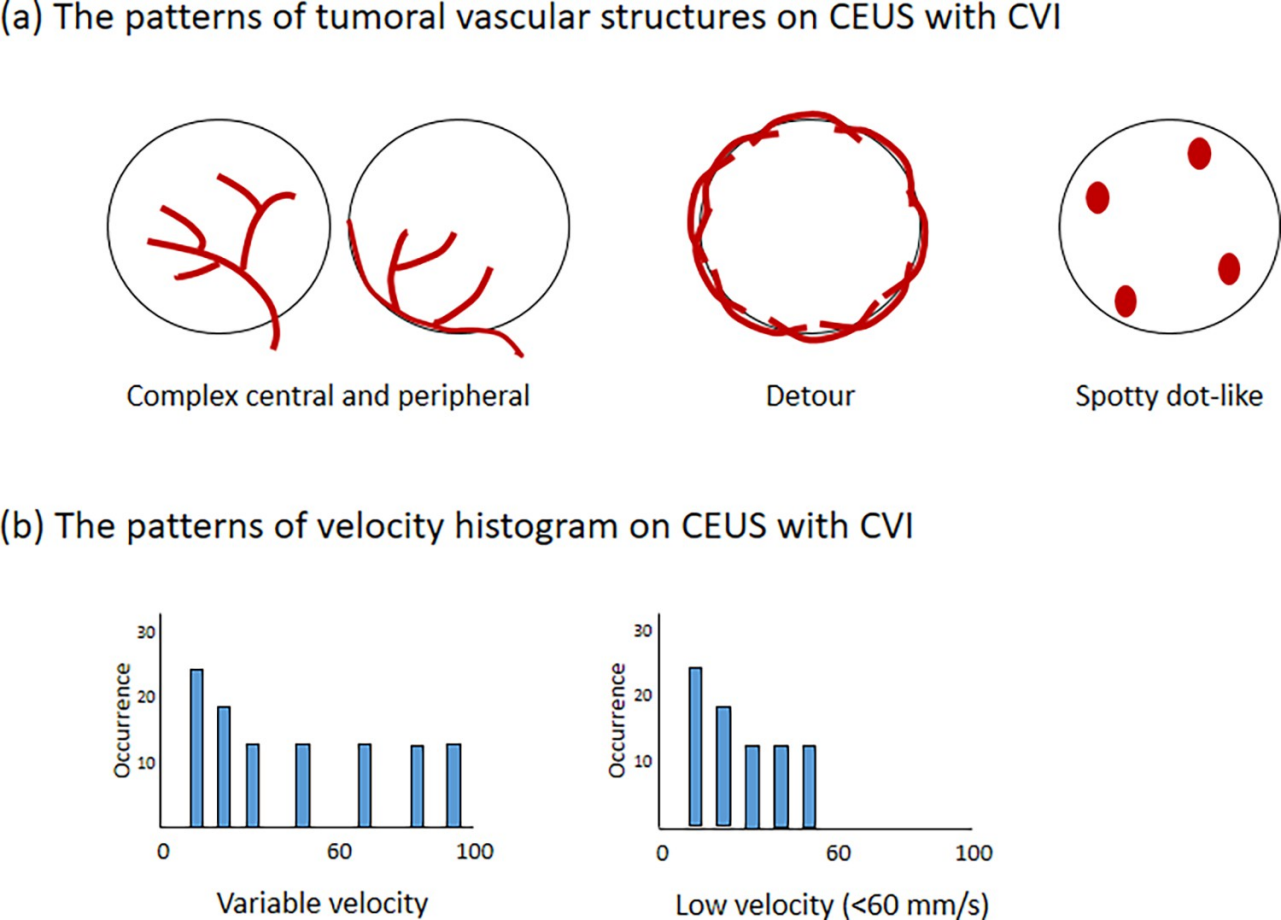
The patterns of tumoral vascular structures (A) and velocity histogram (B) on CEUS with CVI.

The vascular structures of the tumor lesion observed on CVI were categorized into one of four distinct types: central branching, complex central and peripheral (characterized by blood flow entering the tumor mass from the periphery) [15], detour (meandering vessels around the tumor) [16], or spotty dot-like (dots or patches in the tumor) [16] (Fig 2). The distribution patterns of the velocity histogram were classified as low velocity (< 60 mm/s) and variable velocities. In cases of a disagreement, a consensus was reached by discussion with another radiologist (I.J., with 9 years’ experience).

Finally, the two reviewers scored the likelihood of HCC on a 5-point scale (score 1, definitely HCC; 3, indeterminate; 5, definitely non-HCC), taking into account CEUS or CEUS with CVI features and each patient’s underlying liver disease. On CEUS, the reviewers were asked to assign lower scores of 1 or 2 in patients with chronic hepatitis B or liver cirrhosis showing diffuse staining arterial enhancement pattern and late mild washout. The reviewers were instructed to increase the probability of HCC to score 1 or 2 in those at-risk patients exhibiting complex intratumoral and peripheral vessels on CEUS with CVI [15].

### Statistical analysis

All statistical analyses were performed using IBM SPSS Statistics for Windows, v25.0 (IBM Corp., Armonk, New York, USA) and MedCalc Statistical Software v18.9.1 (MedCalc Software bvba, Ostend, Belgium). We used the Chi-square test for comparison of CEUS and CEUS with CVI for the detection of tumoral vascular structures. Arterial enhancement and washout patterns on CEUS and CEUS with CVI were compared among HCC, non-HCC malignancies, and benign tumors using the Chi-square test. Tumoral vascular structures, velocity histograms, and mean velocities on CEUS with CVI were compared among three groups using the Chi-square test and Kruskal-Wallis test, respectively. For comparison of diagnostic performance of CEUS and CEUS with CVI, the area under the receiver operating characteristic curves (AUC) were compared with the z-test. To assess the degree of interobserver agreement, linear-weighted kappa values were calculated: poor, <0.20; fair, 0.20–0.39; moderate, 0.40–0.59; substantial, 0.60–0.79; and almost perfect, >0.80. P-values <0.05 indicated statistical significance.

## Results

### Technical success rate

We could not obtain optimal and sufficient CEUS images (> 30 frames/sec for more than 6 s) in 13 out of 65 cases (the technical success rate = 80.0% [52/65]), and post-processing CVI could not be performed. The reasons for technical failure were a poor sonic window because of overlying basal lung or rib (n = 7), deep location of FLLs away from the probe (n = 5), and respiration-related motion artifacts (n = 1).

### Patients and lesion characteristics

Imaging analysis of CEUS and CVI was performed in 52 patients (the mean age ± standard deviation, 56.3 ± 15.6 years; M:F = 34:18) after excluding 13 cases of technical failure. Twenty-three patients (44.2%) had underlying liver diseases (Table 1). Among the 52 patients, 19 patients had HCC, 13 had non-HCC malignancy (metastasis from colorectal [n = 4], pancreatic [n = 3], breast [n = 1], common bile duct [n = 1], or gallbladder cancer [n = 1], gastrointestinal stromal tumor [n = 1], liver involvement of diffuse large B-cell lymphoma [n = 1], and intrahepatic cholangiocarcinoma [n = 1]), and 20 had benign lesions (FNH [n = 8], hemangioma [n = 5], hepatocellular adenoma [n = 2], angiomyolipoma [n = 2], abscess [n = 2], and intrahepatic reactive lymphoid hyperplasia [n = 1]). The mean lesion size ± standard deviation was 3.5 ± 2.5 cm (range, 1–14).

**Table 1:** Patient enrollment process.

Characteristics	Value
Age (y)	56.3 ± 15.6 (range, 22–81)
Sex	
Men:Women	34 (65.4%):18 (34.6%)
Underlying liver disease	
None	29 (55.8%)
HBV	16 (30.8%)
HCV	3 (5.8%)
Alcohol + HBV	2 (3.8%)
Alcohol	1 (1.9%)
Non-alcoholic steatohepatitis	1 (1.9%)
Liver cirrhosis	
Presence	16 (30.8%)
Absence	36 (69.2%)
Lesion size (cm)	3.5 ± 2.5 (range, 1–14)
Final Diagnosis	
HCC	19 (36.5%)
Non-HCC malignancies	13 (25.0%)
Metastasis from other primary malignancy	12 (23.1%)
Colorectal cancer	4 (7.7%)
Pancreatic cancer	3 (5.8%)
Breast cancer	1 (1.9%)
Gallbladder cancer	1 (1.9%)
Gastrointestinal stromal tumor	1 (1.9%)
Intrahepatic cholangiocarcinoma	1 (1.9%)
Perihilar cholangiocarcinoma	1 (1.9%)
Diffuse large B-cell lymphoma	1 (1.9%)
Benign lesions	20 (38.5%)
FNH	8 (15.4%)
Hemangioma	5 (9.6%)
Hepatocellular adenoma	2 (3.8%)
Angiomyolipoma	2 (3.8%)
Abscess	2 (3.8%)
Intrahepatic reactive lymphoid hyperplasia	1 (1.9%)
Standard of reference	
Pathologic diagnosis	34 (65.4%)
Noninvasive diagnosis of HCC	9 (17.3%)
Typical imaging features of hemangioma (n = 5) and FNH (n = 4) on CECT or MRI	9 (17.3%)

Note.—HBV = Hepatitis B virus; HCV = Hepatitis C virus; HCC = hepatocellular carcinoma; FNH = Focal nodular hyperplasia; CECT = contrast-enhanced CT.

Among 52 lesions, 34 lesions (65.4%) were pathologically confirmed and 9 HCCs (17.3%) were non-invasively diagnosed according to the current guideline [4] (Table 1). Other 9 lesions (17.3%; hemangiomas [n = 5] and FNH [n = 4]) were diagnosed based on typical imaging features on contrast-enhanced CT or MRI with a stability for >2 years. Thirty-one out of 52 patients (59.6%) had contrast-enhanced MRI prior to performing tissue confirmation and/or reaching the final diagnosis.

### Arterial enhancement and washout patterns of HCC, non-HCC malignancy, and benign lesions on CEUS and CEUS with CVI

There were significant differences in arterial enhancement patterns among HCC, non-HCC malignancy, and benign lesions on both CEUS and CEUS with CVI (p = 0.003) (Table 2). HCC (84.2% [16/19]) and benign lesions (65.0% [13/20]) predominantly showed diffuse staining pattern, while peripheral rim pattern was frequent in non-HCC malignancy (53.8% [7/13]) (S1 Table in S1 File).

**Table 2:** Arterial enhancement and washout patterns of HCC, non-HCC malignancies, and benign tumors on CEUS and CEUS with CVI.

	HCC (n = 19)	Non-HCC malignancy (n = 13)	Benign (n = 20)	P value	Interobserver agreement*
Arterial enhancement on CEUS				**0.003**	0.779
Diffuse staining	16 (84.2%)	6 (46.2%)	13 (65.0%)		(0.598–0.960)
Peripheral rim	2 (10.5%)	7 (53.8%)	2 (10.0%)		
Peripheral globular	1 (5.3%)	0	5 (25.0%)		
Arterial enhancement on CEUS with CVI				**0.003**	0.710 (0.518–0.902)
Diffuse staining	16 (84.2%)	6 (46.2%)	13 (65.0%)		
Peripheral rim	2 (10.5%)	7 (53.8%)	2 (10.0%)		
Peripheral globular	1 (5.3%)	0	5 (25.0%)		
Washout				**<0.001**	0.643
Early	1 (5.3%)	10 (76.9%)	1 (5.0%)		(0.470–0.816)
Late and mild	15 (78.9%)	0	4 (20.0%)		
Marked	3 (15.8%)	3 (23.1%)	0		
Absent	0	0	15 (75.0%)		

Note.—HCC = hepatocellular carcinoma; CEUS = contrast-enhanced ultrasonography; CVI = contrast vector imaging.

Significant p-values are indicated in bold.

*Kappa values

HCC, non-HCC malignancy, and benign lesions showed significantly different washout patterns (p<0.001) (Table 2). The majority of HCC lesions (78.9% [15/19]) demonstrated late mild washout, whereas non-HCC malignancy exhibited either early (76.9% [10/13]) or marked (15.8% [3/13]) washout. Benign lesions frequently showed no washout (75.0% [15/20]), and five benign lesions demonstrated late mild washout (20.0% [4/20]; angiomyolipoma, hepatocellular adenoma, hemangioma, and reactive lymphoid hyperplasia) or early washout (5.0% [1/20]; abscess). All benign lesions showing washout except hemangioma were pathologically confirmed.

### Tumoral vascular structures of HCC, non-HCC malignancy, and benign tumors on both CEUS and CEUS with CVI

On CEUS with CVI, HCC, non-HCC malignancies, and bening lesions showed different tumoral vascular structures (p<0.001) (Table 3). Complex intratumoral and peripheral vessels were visualized in all HCC (100% [19/19]), 46.2% (6/13) of non-HCC malignancy, and 70.0% (14/20) of benign tumors. Detour vessels were demonstrated in 53.8% (7/13) of non-HCC malignancy and 10.0% (2/20) of benign tumors. Spotty dot-like vessels were exclusively shown in benign tumors (20% [4/20]); all four lesions were hemangiomas.

**Table 3:** Comparison of tumoral vascular structures of HCC, non-HCC malignancy, and benign tumors on CEUS and CEUS with CVI.

		HCC (n = 19)	Non-HCC malignancy (n = 13)	Benign (n = 20)	P-value
CEUS	Complex intratumoral & peripheral	8 (42.1%)	2 (15.4%)	3 (15.0%)	**<0.001**
	Detour	0	7 (53.8%)	0	
	Spotty dot-like	0	0	4 (20.0%)	
	Central branching	0	0	0	
	Non-evaluable	11 (57.9%)	4 (30.8%)	13 (65.0%)	
CEUS + CVI	Complex intratumoral & peripheral	19 (100.0%)	6 (46.2%)	14 (70.0%)	**<0.001**
	Detour	0	7 (53.8%)	2 (10.0%)	
	Spotty dot-like	0	0	4 (20.0%)	
	Central branching	0	0	0	
	Non-evaluable	0	0	0	

Note.—CEUS = contrast-enhanced ultrasonography; CVI = contrast vector imaging.

Significant p-values are indicated in bold.

On CEUS, complex intratumoral and peripheral vessels were demonstrated in 42.1% (8/19) of HCC, 15.4% (2/13) of non-HCC malignancy, and 15.0% (3/20) of benign tumors (p<0.001) (Table 3). Detour vessels were visualized in 15.8% (3/19) of HCC, 61.5% (8/13) of non-HCC malignancy, and none of benign tumor (p = 0.021). Spotty dot-like vessels were exclusively shown in one hemangioma lesion from benign group (20.0% [4/20]).

The detectability of tumoral vascular structures was significantly higher in CEUS with CVI, compared to CEUS alone (46.2% [24/52] vs. 100.0% [52/52], p<0.001) (Table 3).

### Comparison of velocity histogram patterns and mean velocity of HCC, non-HCC malignancy, and benign tumors on CEUS with CVI

**Fig 3:**
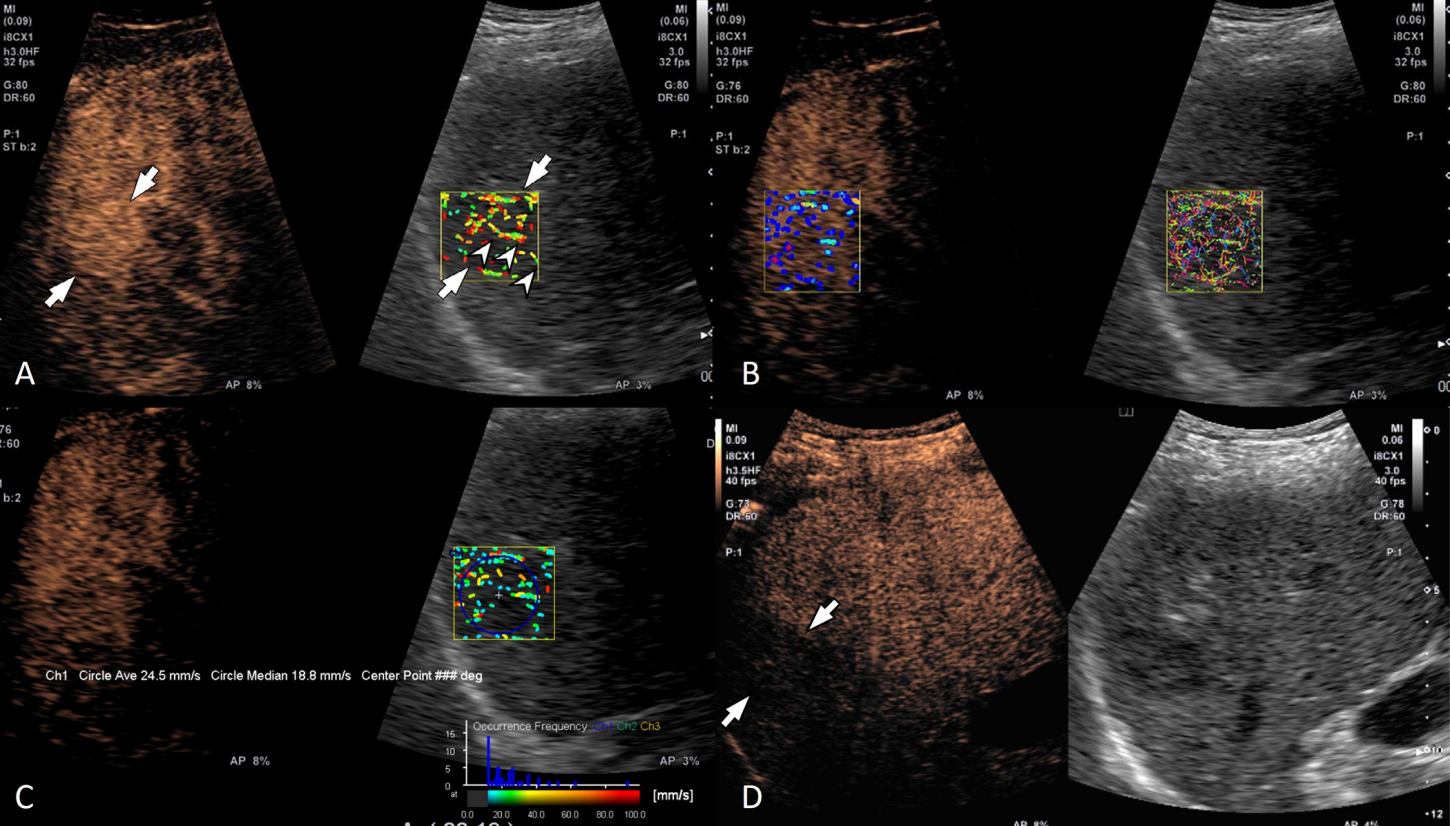
A 63-year-old man with hepatitis C viral infection-related liver cirrhosis and a 3 cm hepatocellular carcinoma in the liver segment 7. (A) Contrast-enhanced ultrasound (left) and velocity mode of contrast vector imaging (CVI) (right) show a diffuse staining pattern (arrows) during the arterial phase. A penetrating vessel (arrowheads) is well visualized on CVI. (B) Both velocity variance mode (left) and trace mode (right) of CVI show a diffuse staining pattern in the arterial phase. (C) Histogram analysis showed variable velocity and the mean velocity was 24.5 mm/s. (D) The lesion (arrows) showed a late mild washout pattern.

**Fig 4:**
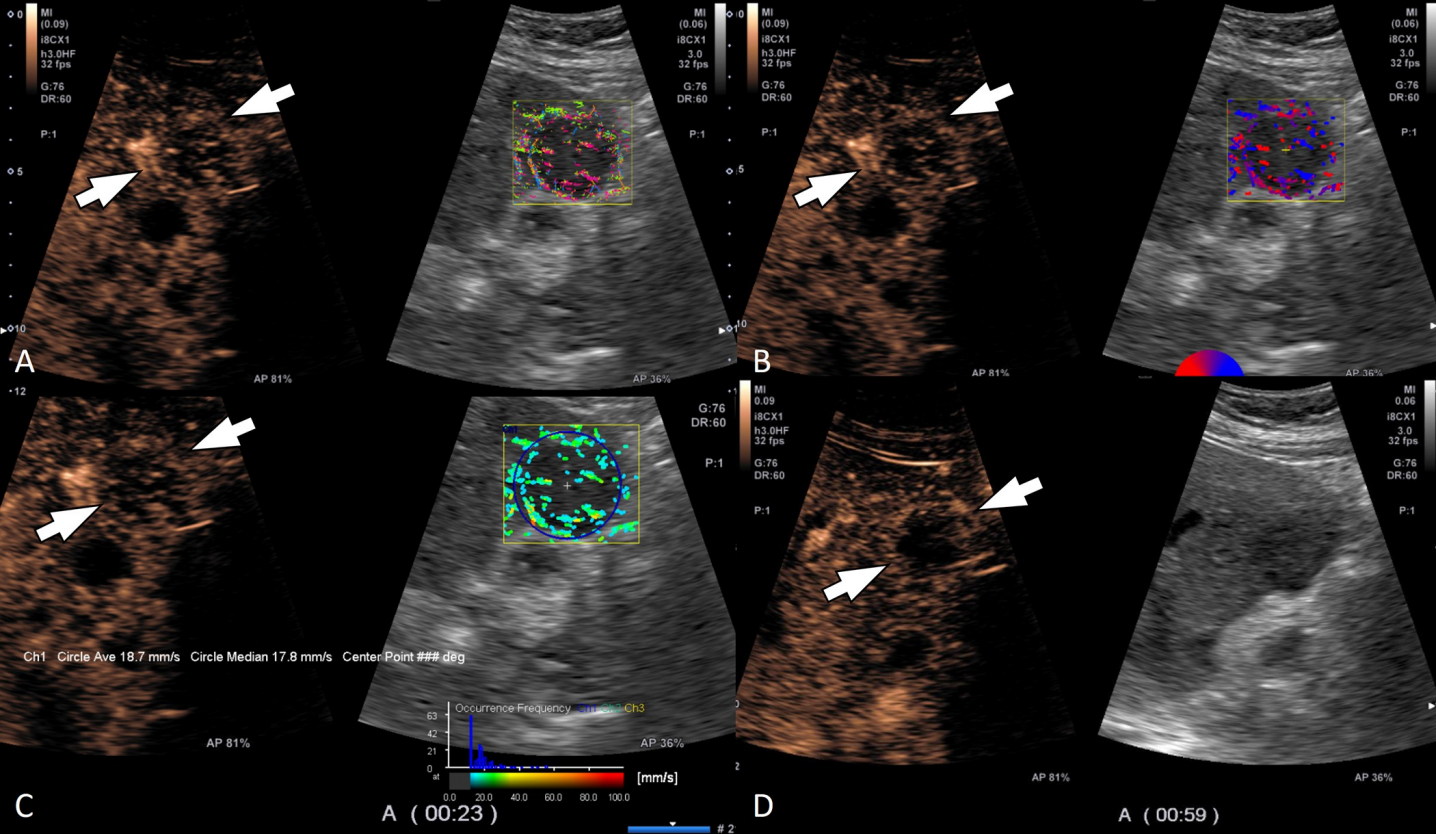
A 53-year-old woman with liver metastasis from GB cancer. (A) Contrast-enhanced ultrasound (left) and trace mode of contrast vector imaging (CVI) shows a peripheral rim pattern during the arterial phase. (B) Tumoral vascular structure was assessed as having both penetrating and detour patterns on velocity variance mode of CVI (right). (C) Histogram analysis showed low velocity less than 60 mm/s and the mean velocity was 18.7 mm/s. (D) The lesion showed early washout within 1 min.

**Fig 5:**
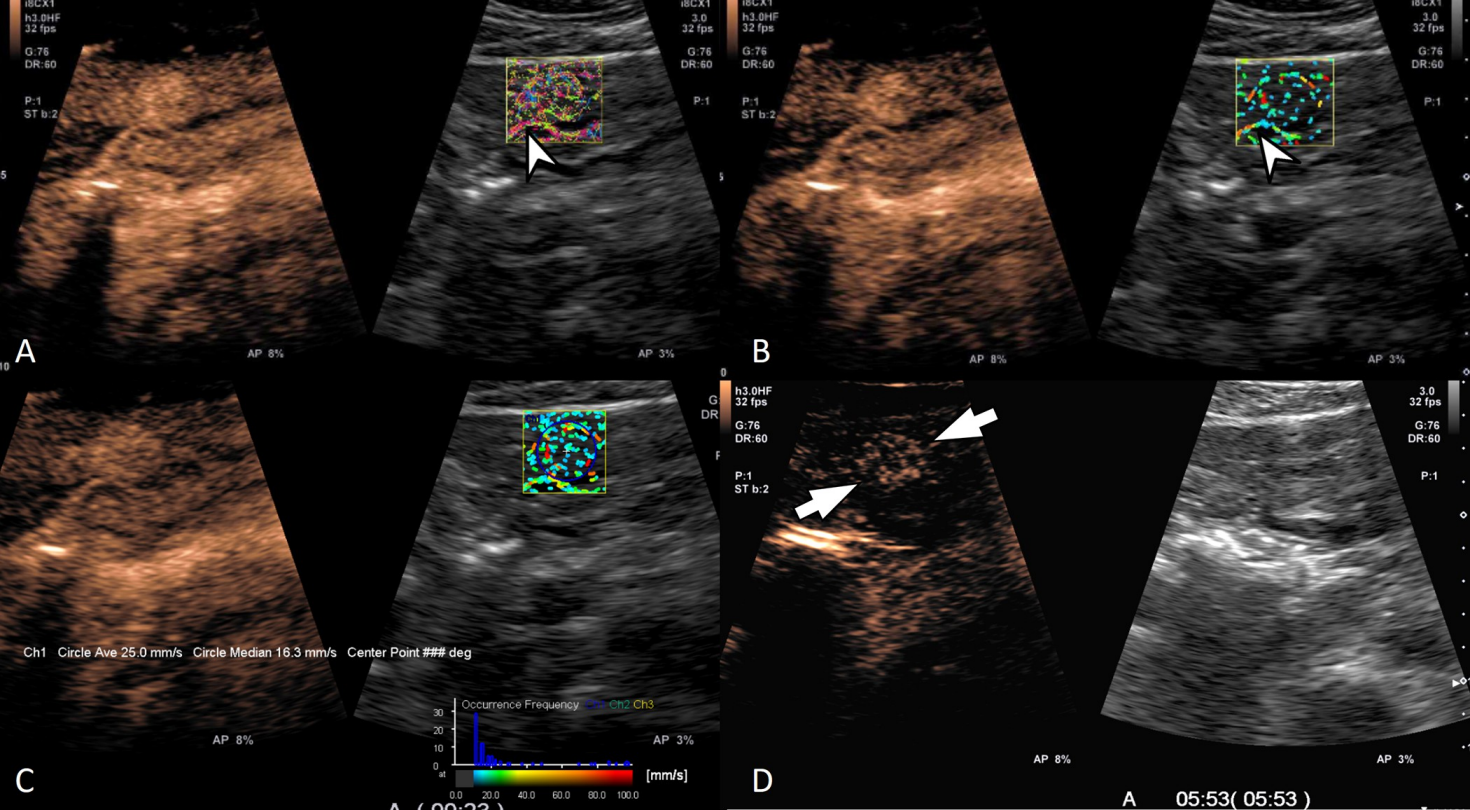
A 31-year-old man with focal nodular hyperplasia in the liver segment 3. (A) Contrast-enhanced ultrasound (left) and trace mode of contrast vector imaging (CVI) show a diffuse staining pattern during the arterial phase. (B) Velocity mode of CVI (right) shows penetrating vessels (arrowhead). (C) Histogram analysis shows variable velocities of intratumoral flow and the measured mean velocity with CVI was 24.5 mm/s. (D) The lesion does not show a washout until 5 min after intravenous administration of contrast agent.

Regarding the velocity histogram pattern, HCC (73.7% [14/19]) and benign tumors (60.0% [12/20]) predominantly showed variable velocity and non-HCC malignancy (53.8% [7/13]) frequently showed a low velocity; there were no significant differences among three groups (p = 0.280) (Table 4). Quantitative analyses showed that the mean velocity of HCC (26.3 mm/s) was the highest among the three groups, while that of non-HCC malignancy (20.6 mm/s) was the lowest (p<0.001). Representative cases are shown as Figs 3–5.

**Table 4:** Comparison of velocity histogram patterns and mean velocity of HCC, non-HCC malignancy, and benign tumors on CEUS with CVI.

	HCC (n = 19)	Non-HCC malignancy (n = 13)	Benign (n = 20)	P-value
Velocity histogram				0.280
Variable velocity	14 (73.7%)	6 (46.2%)	12 (60.0%)	
Low velocity (< 60 mm/s)	5 (26.3%)	7 (53.8%)	8 (40.0%)	
Mean velocity (mm/s)	26.3 ± 9.9	20.6 ± 2.3	24.7 ± 7.3	**<0.001**

Note.—HCC = hepatocellular carcinoma; CEUS = contrast-enhanced ultrasonography; CVI = contrast vector imaging.

Significant p-values are indicated in bold.

### Comparison of diagnostic performance between CEUS and CEUS with CVI

Regarding the determination of HCC probability based on both imaging features and patients’ risk factors for HCC, CEUS with CVI showed higher AUC, compared to CEUS alone in both reviewers (0.851 vs. 0.963, p = 0.005 for reviewer 1; 0.853 vs. 0.982, p = 0.023 for reviewer 2) (S2 Table in S1 File). No significant differences were found in sensitivity of CEUS and CEUS with CVI in both reviewers (73.7% [14/19] vs. 94.7% [18/19], p = 0.125 for reviewer 1; 78.9% [15/19] vs. 100% [19/19], p = 0.125 for reviewer 2).

### Inter-observer agreement

On CEUS and CEUS with CVI, inter-observer agreement for arterial enhancement pattern and washout pattern was substantial (κ = 0.779 and 0.643, respectively) (Table 2). Inter-observer agreement for tumoral vascular structures was moderate on both CEUS (κ = 0.457) and CEUS with CVI (κ = 0.502). On CEUS with CVI, the pattern of the velocity histogram showed almost perfect (κ = 0.871) inter-observer agreement.

## Discussion

In this study, an augmented review of CVI demonstrated enhanced detection capabilities for intratumoral vascular structures, compared to CEUS alone. This integrated approach yielded superior diagnostic efficacy in ascertaining the likelihood of HCC probability in comparison to CEUS alone. Furthermore, HCC, non-HCC malignancies, and benign tumors demonstrated different tumoral vascular structures on CEUS with CVI; complex intratumoral and peripheral vessels were visualized in all HCC lesions, while detour vessels were predominantly demonstrated in non-HCC malignancies, and spotty dot-like vessels were exclusively visualized in hemangiomas. Our quantitative analyses showed that the mean velocity of HCC was the highest among the three groups, while that of non-HCC malignancy was the lowest. Although CEUS can better demonstrate arterial enhancement patterns of FLLs than CT and MRI owing to its high temporal resolution [7, 17], analysis of tumor-specific vascularization patterns on CEUS has been overlooked. The reason for this negligence might be explained by the fact that those vascular structures are briefly visualized in the early arterial phase for a few seconds after contrast bubble arrival [10], which quickly mix with intratumoral stromal enhancement. In the present investigation, the enhanced visualization of tumoral vessels on CEUS with CVI may ascribed to the utilization of a high frame rate during CEUS acquisition, in conjunction with the bubble tracking feature provided by CVI. This approach facilitated the visualization of the continuous vascular course, independently of the lesion enhancement. Taking into account that CVI was reconstructed using routine CEUS images, it can be inferred that CVI may serve as an auxiliary diagnostic tool to support CEUS in the characterization of FLLs, rather than serving as an independent diagnostic modality.

Recent research has demonstrated that advanced power Doppler imaging (PDI)-based US techniques such as superb microvascular imaging or microvascular flow imaging could depict tumoral vessels with higher sensitivity than PDI [18, 19]. Although those techniques could provide vascular information without contrast media injection, they are not without limitations. PDI-related artifacts, such as “blooming” or “flash” artifacts, are particularly prevalent in lesions situated at the hepatic dome or left lobe. Furthermore, the accuracy of these techniques is limited when assessing deep-seated lesions [19]. As CVI, which is obtained from CEUS data, is free from those artifacts, it can be used for FLLs on the left lobe or hepatic dome.

In addition, CVI furnished qualitative and quantitative details on intratumoral vascular flows of FLLs. All HCC showed complex central and peripheral vessels with the highest mean velocity among the three groups on CEUS with CVI. Our results are similar to the results of color Doppler US showing turbulence flow and a high-frequency peak [20] and may reflect hemodynamics of HCC having neovascularized arteries [21] with intratumoral vascular shunts [20, 22, 23]. Benign FLLs showed variable patterns of tumoral vascular structures and velocity histograms on CVI, which were also reported in previous studies on Doppler ultrasound [20, 24, 25]. In our study, hemangiomas commonly displayed a pattern of spotty, dot-like vessels and consistently demonstrated low velocity as observed on the histogram. However, benign solid hypervascular tumors such as FNH or hepatic adenomas often showed complex central and peripheral vascular pattern on CVI. Although both HCC and FNH showed similar vascular patterns on CVI, the presence of risk factor for HCC and absence of washout facilitate differentiation between the two lesions. Nevertheless, in cases where imaging findings are inconclusive, biopsy remains the definitive standard for diagnosis. Consequently, while the additional benefits of CVI might seem limited, vascular patterns can serve as ancillary imaging features when the enhancement patterns of CEUS are ambiguous.

While Doppler ultrasound is capable of measuring blood flow velocity, the color Doppler examination is subject to interobserver variation and angle dependency [26]. However, utilizing CVI has the potential to mitigate these issues, leading to a reduction in interobserver discrepancies and enhancing the accuracy of flow velocity measurements.

There are several limitations in our study. First, a majority of non-HCC malignancies were hypovascular metastasis, but hypervascular metastases were not included. This may have caused the overestimation of the performance of CEUS with CVI in differentiating HCC from non-HCC. However, given that HCC are more prevalent than hypervascular metastases, especially in patients with liver cirrhosis, the likelihood of this scenario is minimal. Second, most HCCs in our study were diagnosed using the non-invasive imaging criteria, not by histopathologic analysis, thereby precluding the confirmation of tumoral vascular structures through pathology. Third, the radiologists were not blinded to patients’ risk factors for HCC, which might have affected diagnostic performances. However, it reflects routine clinical practice in which the presence or absence of patients’ risk factors for HCC is paramount for diagnostic pathways. Fourth, it is important to note that the applicability of our findings may be restricted due to the fact that CVI can only be performed using a high-end ultrasound unit from a specific vendor. Next, complex intratumoral and peripheral vessels were frequently present in HCC as well as benign tumors, thus there can be limitation in differentiating HCC from benign tumors. Last, although our study demonstrated that adding CVI findings to CEUS could provide additional value for improving diagnostic performance in making HCC diagnosis in patients at risks, this was a single center study having a relatively small number of study patients. Furthremore, given the nature of our patient enrollment process, there is a possibility that our dataset may include a disproportionate number of atypical FLLs, particularly those cases where CEUS was performed for problem-solving purposes. Thererfore, a further large-scale multicenter study is warranted to demonstrate its clinical value for noninvasive diagnosis of HCC in patients at risks.

In conclusion, the application of CEUS in conjunction with CVI demonstrated a higher AUC, albeit with similar sensitivity for assessing the likelihood of HCC, compared to CEUS alone. Notably, our findings revealed distinct variations in tumoral vessels and mean velocity among HCC, non-HCC malignancies, and benign tumors, further highlighting the diagnostic potential of the CEUS-CVI methodology.

## Supporting information

S1 File (DOCX)
