# Supplementary material for: Contrast vector imaging for differential diagnosis of focal liver lesions: Analysis of tumoral vascular structures and flow characteristics
Source: PLoS One. 2024 Dec 3;19(12):e0314263. doi: 10.1371/journal.pone.0314263 (PMC11614252; doi:10.1371/journal.pone.0314263)
Supplement: S1 File — (DOCX) [file pone.0314263.s001.docx]

Supplementary Fig. 1. Seven display types of CEUS with CVI


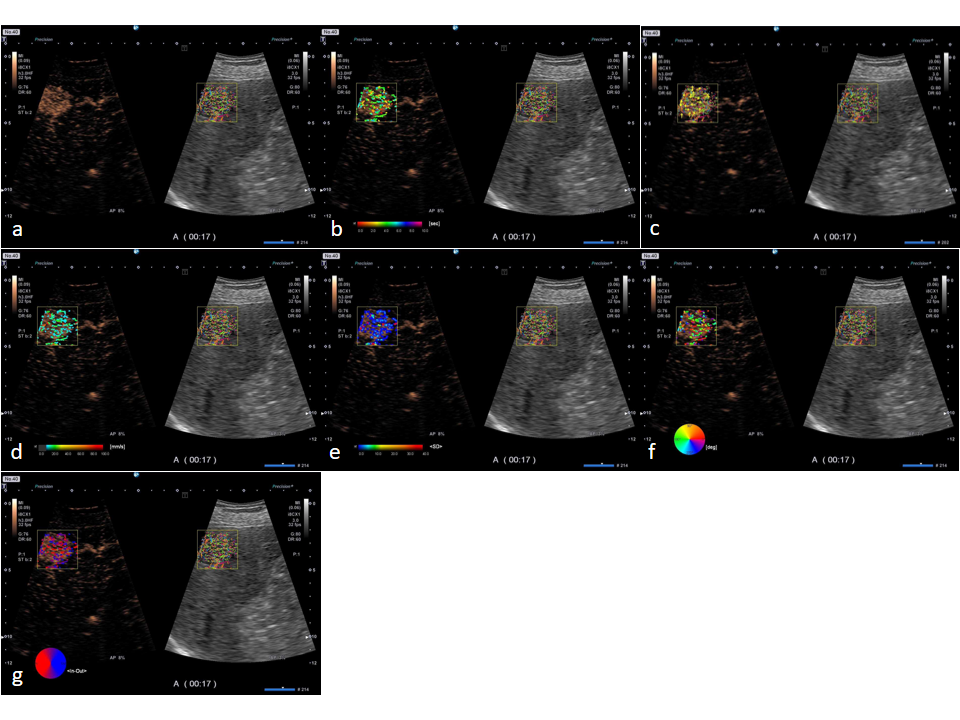


1. CEUS (left) and trace map (right). The trace map visualizes all the bulbbles that were detected.
2. Arrival time (left) and trace (right) maps: The arrival time visualize color-coded information regarding arrival time (sec) of each bubble.
3. Arrow (left) and trace (right) maps: The arrow map visualizes all the bubbles that were detected with their movements in arrows.
4. Velocity (left) and trace (right) maps: The velocity map shows color-coded information regarding each bubble’s velocity, and provides mean and median velocities of bubbles in a region-of-interest.
5. Velocity variance (left) and trace (right) maps: The velocity variance map calculates variance of diffferent bubble’s velocities moving along the same route and shows it as a color-coded map.
6. Direction (left) and trace (right) maps: The direction map visualize each bubble’s direction from the center of a region-of-interest as a color-coded map.
7. Direction center (left) and trace (right) maps: The direction center map visualizes whether a bubble is moving into the center (red) of a region-of-interest or moving out of the center (blue).

Supplementary Table 1. Comparison of arterial enhancement patterns on CEUS and CEUS with CVI

| CEUS | CEUS with CVI | HCC (n=19) | Non-HCC malignancy (n=13) | Benign (n=20) | Total (n=52) |
| --- | --- | --- | --- | --- | --- |
| Diffuse staining  (n=35) | Diffuse staining  Peripheral rim  Peripheral globular | 15  1  0 | 5  1  0 | 13  0  0 | 33 (94.3%)  2 (5.7%)  0 |
| Peripheral rim  (n=11) | Diffuse staining  Peripheral rim  Peripheral globular | 1  1  0 | 1  6  0 | 0  2  0 | 2 (18.2%)  9 (81.8%)  0 |
| Peripheral globular (n=6) | Diffuse staining  Peripheral rim  Peripheral globular | 0  0  1 | 0  0  0 | 0  0  5 | 0  0  6 (100%) |

Note. —CEUS = contrast-enhanced ultrasonography; CVI = contrast vector imaging; HCC = hepatocellular carcinoma.

Supplementary Table 2. Determination of HCC probability on CEUS and CEUS with CVI by two reviewers

|  | |  | CEUS | | | | | |
| --- | --- | --- | --- | --- | --- | --- | --- | --- |
|  |  | Score | 1 | 2 | 3 | 4 | 5 | Total |
| CEUS with CVI | Reviewer 1 | 1 | 14 (26.9%) | 0 | 0 | 0 | 0 | 14  (26.9%) |
|  |  | 2 | 0 | 0 | 1 (1.9%) | 2  (3.8%) | 1  (1.9%) | 4  (7.7%) |
|  |  | 3 | 0 | 0 | 0 | 0 | 0 | 0 |
|  |  | 4 | 2  (3.8%) | 2  (3.8%) | 0 | 9  (17.3%) | 0 | 13  (25.0%) |
|  |  | 5 | 0 | 0 | 3  (5.8%) | 1  (1.9%) | 17  (3.3%) | 21  (40.4%) |
|  |  | Total | 16  (30.8%) | 2  (3.8%) | 4  (7.7%) | 12  (23.1%) | 18  (34.6%) | 52  (100.0%) |
|  | Reviewer 2 | 1 | 15 (28.8%) | 0 | 1  (1.9%) | 0 | 0 | 16  (30.8%) |
|  |  | 2 | 0 | 0 | 0 | 1  (1.9%) | 3  (5.8%) | 4  (7.7%) |
|  |  | 3 | 0 | 0 | 0 | 0 | 0 | 0 |
|  |  | 4 | 0 | 0 | 3  (5.8%) | 14  (26.9%) | 2  (3.8%) | 19  (36.5%) |
|  |  | 5 | 0 | 0 | 1  (1.9%) | 1  (1.9%) | 11 (21.2%) | 13  (25.0%) |
|  |  | Total | 15 (28.8%) | 0 | 5  (9.6%) | 16  (30.8%) | 16  (30.8%) | 52  (100.0%) |

Note. — HCC = hepatocellular carcinoma; CEUS = contrast-enhanced ultrasonography; CVI = contrast vector imaging; score 1, definitely HCC; 2, probably HCC; 3, indeterminate; 4, probably non-HCC; 5, definitely non-HCC.
